# Osteosarcoma of the Mandible in an Elderly Patient

**DOI:** 10.1155/2022/2622551

**Published:** 2022-03-17

**Authors:** Isao Hoshi, Ryosuke Abe, Kei Onodera, Yu Ohashi, Tadashi Kawai, Ikuya Miyamoto, Toshimi Chiba, Yasunori Takeda, Hiroyuki Yamada

**Affiliations:** ^1^Division of Oral and Maxillofacial Surgery, Department of Reconstructive Oral and Maxillofacial Surgery, School of Dentistry, Iwate Medical University, Morioka 020-8505, Japan; ^2^Department of Oral and Maxillofacial Surgery, Iwate Prefectural Central Hospital, Morioka 020-0066, Japan; ^3^Division of Internal Medicine, Department of Oral Medicine, Iwate Medical University, Morioka 020-8505, Japan; ^4^Division of Clinical Pathology, Department of Oral and Maxillofacial Reconstructive Surgery, School of Dentistry, Iwate Medical University, Morioka 020-8505, Japan

## Abstract

Osteosarcoma is a malignant tumor in which the cancerous cells produce an osteoid matrix or mineralized bone. Jaw bones are affected in 6% of all osteosarcomas and are the fourth most common site of origin. Surgical treatment of osteosarcoma in elderly patients is rarely reported. Here, we report successful treatment of osteosarcoma arising in the mandible of a 90-year-old man. The patient was referred to our institution for diagnosis and treatment of an oral lesion. Intraoral examination revealed that a hard mass measuring 35 × 27 mm was located on the floor of the oral cavity, attached to the bone, and its growth displaced the tongue posteriorly. Therefore, he experienced difficulty in speech and swallowing. Biopsy of the mandibular mass was suspicious for chondrosarcoma. Preoperative examination did not detect critical risks for general anesthesia or surgery. Based on a clinical diagnosis of a malignant bone tumor of the mandible, segmental mandibular resection with reconstruction using a titanium plate was performed. Surgical site infection occurred on postoperative day 12, which was resolved by drainage, local irrigation, and administration of antibiotics. There was no delirium or cardiovascular or pulmonary complications. Surgery resolved the patient's difficulties in speech and swallowing. There was no evidence of tumor recurrence or metastasis 4 years after surgery. This case showed that it was not necessary to exclude surgical treatment merely because the patient was 90 years old. Indications for surgery should be determined individually to improve the patient's quality of life.

## 1. Introduction

Osteosarcoma is a malignant tumor in which the cancerous cells produce an osteoid matrix or mineralized bone [1]. Jaw bones are affected in 6% of all osteosarcomas and are the fourth most common site of origin, following the femur, tibia, and humerus [2]. Osteosarcoma of the jaw (OSJ) tends to occur in the mandible twice as often as in the maxilla [3, 4]. No gender difference for the incidence of OSJ has been reported [2]. OSJ develops in older age and shows good prognosis when compared with that of tumors in the long bones [2]. Therefore, OSJ is considered to be distinct from osteosarcoma arising in the long bones [4]. The standard treatment for osteosarcoma of the long bones is surgery with perioperative chemotherapy [5]; however, it is not similarly applicable for OSJ [2]. A review of the English language literature revealed 214 cases of OSJ diagnosed over 40 years in patients with a maximum age of 83 years [4]. In another retrospective cohort study of 541 cases of OSJ, the maximum age was 91 years [6]. The mean age of patients with OSJ is reported to be 39–42 years; therefore, surgical treatment of osteosarcoma in elderly patients is rarely reported [3, 4, 6]. Here, we report successful treatment of osteosarcoma arising in the mandible in a 90-year-old man.

## 2. Case Presentation

**Figure 1:**
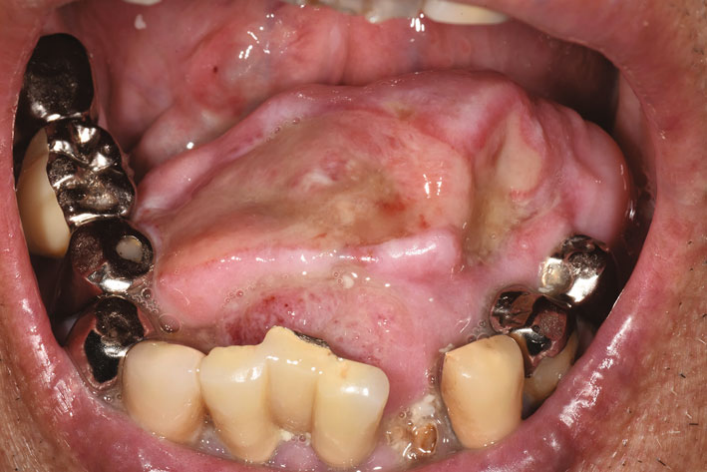
Intraoral photograph showing a large mass occupying the oral floor. The overlying oral mucosa is partially ulcerated.

**Figure 2:**
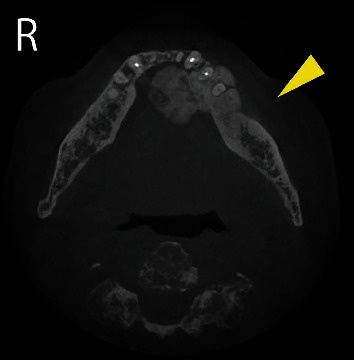
Cone-beam computed tomography showing the mass connected to the cortical bone of the mandible. The mass spread to the left mandibular body (arrowhead).

**Figure 3:**
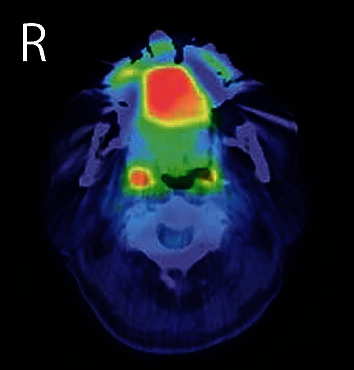
Positron emission tomography/computed tomography showing abnormal accumulation of fluorodeoxyglucose in the oral mass (maximum standardized uptake value 7.2).

**Figure 4:**
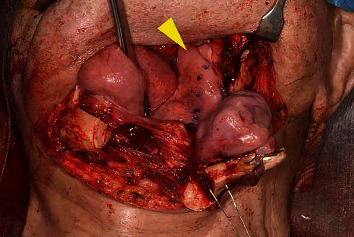
Intraoperative photomicrograph showing surgical safety margin of about 10 mm set in the normal-looking tissue (arrowhead).

**Figure 5:**
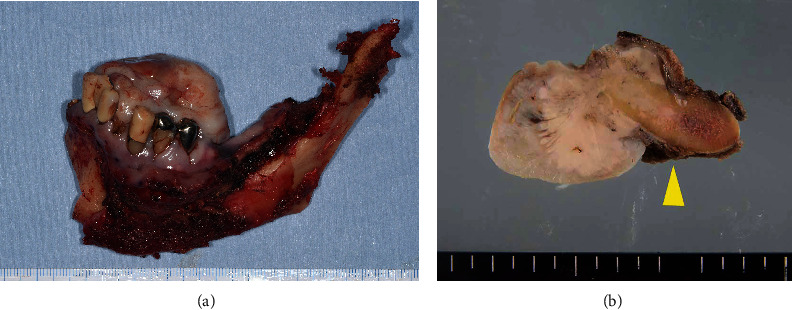
(a) Resected mandibular specimen revealing dominant exophilic proliferation. (b) Cut surface of the tumor after fixing with formalin showing infiltration to the alveolar bone (arrowhead).

**Figure 6:**
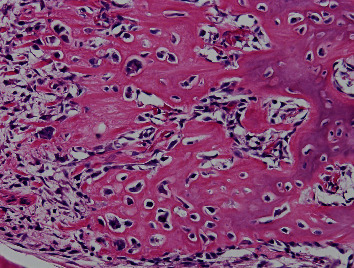
Photomicrograph showing trabeculae with low calcification spread radially in the periphery of the tumor. Tumor cells with mild atypia are present densely, with partial cartilage ossification (hematoxylin-eosin stain, ×400).

**Figure 7:**
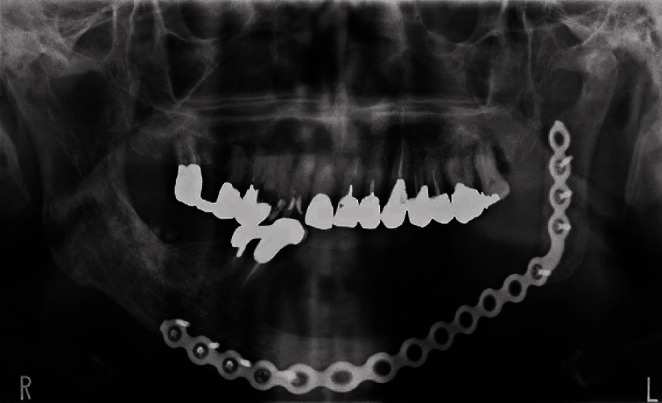
Postoperative panoramic X-ray showing the reconstructed mandible with a titanium plate.

A 90-year-old man was referred to our institution in May 2017 for diagnosis and treatment of an oral lesion. He had first noticed the lesion approximately 1 year earlier. The lesion grew gradually and showed rapid enlargement in the last 6 months. The patient's medical history included atrial fibrillation treated with warfarin, hyperlipidemia, resected rectal cancer, and internal carotid artery stenosis treated by carotid endarterectomy. All of these conditions were followed up and controlled by the patient's physicians. He had smoked cigarettes from age 23 to 48 years, resulting in a Brinkman index of 3,000. His family history was unremarkable. Intraoral examination revealed that a hard mass measuring 35 × 27 mm was located on the floor of the oral cavity and attached to the bone. The overlying oral mucosa was partially ulcerated. The growth of the mass displaced the tongue posteriorly, resulting in a Mallampati score of IV (Figure 1); therefore, he experienced difficulty with speech and swallowing. There was no evidence of cranial nerve paralysis or cervical lymphadenopathy. Cone-beam computed tomography (CT) (3D Accuitomo F17; Morita, Kyoto, Japan) revealed that the mass was connected to the cortical bone of the mandible. The mass was inhomogeneous and spread to the left mandibular body (Figure 2). Magnetic resonance imaging was performed with a 3.0-Tesla system (MR750; General Electric Company, Boston, MA, USA). On T1- and T2-weighted axial images, the mass showed low signal intensity relative to muscle. Positron emission tomography (PET)/CT was performed with a Discovery PET/CT 600 scanner (General Electric Company). The image showed abnormal accumulation of fluorodeoxyglucose in the mandibular lesion (maximum standardized uptake value: 7.2) (Figure 3). There was no abnormal accumulation of fluorodeoxyglucose in any other organ. Biopsy of the mandibular mass was suspicious for chondrosarcoma. The American Society of Anesthesiologists (ASA) physical status was class 3. Echocardiography revealed an ejection fraction of 64% without ischemic or valvular heart disease. Spirography revealed that forced expiratory volume in 1 second was 64.8%, suggesting slight obstructive ventilation disorder. Magnetic resonance angiography of the internal carotid artery revealed no evidence of restenosis after carotid endarterectomy. Preoperative examination did not detect critical risks for general anesthesia and surgery, although their duration was reduced as much as possible. No method other than surgical treatment could have resolved his chief complaint. Heparin bridging was scheduled for atrial fibrillation treated with warfarin before surgery. Based on a clinical diagnosis of a malignant bone tumor of the mandible (cT1N0M0), segmental mandibular resection with reconstruction using a titanium plate was performed in June. Although the Mallampati score was IV, tracheal intubation was not difficult without trismus. During the operation, a surgical safety margin of about 10 mm was set in the normal-looking tissue. First, we reached the inferior border of the mandible through cervical incision. Next, we made an intraoral incision on the buccal gingiva. By resecting soft tissue around the tumor, we connected the oral wound to the cervical one. This procedure exposed the lateral surface of the tumor with surrounding soft tissue. The medial border was cut by the surgical saw, and the mandible was swung to the left side to visualize the inner surface of the tumor. By directly viewing the tumor attached to the bone, the oral floor was incised (Figure 4). Resection of the tumor with surrounding normal soft tissue was performed backward, and the inferior alveolar nerve and vascular bundles were ligated and cut. Finally, mandibular segmental resection was completed by cutting the mandibular ramus at the distal border. After removal of the tumor, the mandible was reconstructed using a titanium plate that was prepared preoperatively based on a 3D mandibular model. Operation time was 5 h, and the amount of bleeding was 389 g. After the operation, the patient received frequent nursing care. The resected mandibular specimen revealed dominant exophilic proliferation (Figure 5(a)), and the cut surface of the tumor after fixing with formalin showed infiltration to the alveolar bone (Figure 5(b)). Histopathological examination revealed that trabeculae with low calcification spread radially in the periphery of the tumor. Tumor cells with mild atypia were present densely with partial cartilage ossification (Figure 6). The surgically resected margins were negative for tumor. The final histopathological diagnosis was low-grade osteosarcoma (stage IA). Surgical site infection occurred on postoperative day 12, which was resolved by drainage, local irrigation, and administration of antibiotics. There was no delirium or cardiovascular or pulmonary complications. Surgery resolved the patient's speech and swallowing difficulties. He continued to come to our hospital alone on foot for regular clinical check-up. There was no evidence of tumor recurrence or metastasis 4 years after surgery by evaluation with panoramic radiography (Figure 7) and chest X-ray.

## 3. Discussion

Surgery in the oldest patients has various complications, including cardiovascular and pulmonary complications, surgical site infection, and delirium. The cardiovascular risk index is a newly proposed method for preoperative cardiovascular evaluation [7]. The index is based on 6 easily acquired data elements (age > 75 years, history of heart disease, symptoms of angina or dyspnea, hemoglobin < 12 mg/dl, vascular surgery, and emergency surgery) that can strongly predict the 30-day risk of postoperative cardiovascular events in patients undergoing noncardiac surgery. If the present case is evaluated using this index, age and history of heart disease are 2 positive elements, resulting in an intermediate risk retrospectively. Postoperative pulmonary complications are closely related to the surgical site. Their incidence is highest among patients who undergo cardiothoracic surgery (5.3%) and lowest in orthopedic and urological surgery (0.7%) [8]. Head and neck surgery carries an intermediate risk, and advanced age is an important independent predictor of postoperative pulmonary complications in noncardiothoracic surgery [9]. Cigarette smoking indicates a modest increase in complication risk [9]. At least 2 months of smoking cessation is recommended to reduce the risk of increased intraoperative sputum volume [10]. In the present case, a high Brinkman index was documented; however, smoking cessation had been continued for >40 years. Delirium is an acute fluctuating alteration of mental state, reduced awareness, and disturbance of attention that may be triggered by acute medical illness, surgery, trauma, or drugs [11]. After head and neck surgery, the incidence of postoperative delirium is reported to be 19.26% [12]. The occurrence of postoperative delirium is significantly related to increased age, mental health status, ASA score, and drug dependence [13]. In the present case, good mental condition, absence of drug dependence, and frequent nursing care might have contributed to the absence of delirium. Consequently, major complications except for surgical site infection were avoided. The most equivocal differential diagnosis of low-grade osteosarcoma is benign fibro-osseous lesions. The presence of proliferative infiltration is the main histopathological finding to exclude fibro-osseous lesions. Moreover, immunohistochemically positive staining for murine double-minute type 2 and cyclin-dependent kinase 4 is useful to distinguish osteosarcoma from benign fibro-osseous lesions [14]. Although immunohistochemical analysis was not performed in the present case, the histopathological finding of infiltration of tumor cells to the alveolar bone, with cartilage ossification, was consistent with the diagnosis of osteosarcoma.

Osteosarcoma is also a common secondary malignancy following radiotherapy of a primary malignant tumor [15]. In the present case, irradiation to the head and neck was not performed previously. The surgically resected specimen did not clearly show the preexisting mandibular lesion. PET showed no abnormal accumulation of fluorodeoxyglucose in any other organ except for the mandible. Therefore, the present case was classified as primary rather than secondary osteosarcoma. The standard treatment for high-grade osteosarcoma is surgery with pre- and postoperative chemotherapy [5]. However, low-grade OSJ can typically be cured by complete resection [4]. In the present case, the result of preoperative biopsy was suspected chondrosarcoma. Because the standard treatment for chondrosarcoma is surgery [5], we selected surgery without preoperative chemotherapy. The final histopathological diagnosis of low-grade osteosarcoma, complete surgical removal of the tumor, and the patient's age of 90 years prompted us to select postoperative observation without chemotherapy. As a result, the patient survived for 4 years without tumor recurrence or metastasis. The vascularized fibula using a microsurgical technique is the most popular free flap for mandibular reconstruction [16] and should be selected if the patient is not elderly. However, this method requires a long operation time. In the present case, the duration of general anesthesia and surgery was reduced as much as possible. Therefore, minimally required operation was selected. Fortunately, soft tissue deficiency after segmental mandibular resection was small because of the exophilic nature of the tumor. The presence of mislocalized cytoplasmic p27 is reported to be associated with higher metastatic status and poorer survival of osteosarcoma [17]. As clinical factors, proximal location of osteosarcoma in the long bone of an extremity portends a worse clinical outcome than tumors located in the distal long bones [18]. The prognostic factors for OSJ have been reported in a few studies [4, 6]. The biomarker melanoma-associated antigen-A failed to predict the prognosis of OSJ [3]. The clinical factors of the lower tumor stage [6], surgery performed [6], clear surgical margin [4], and earlier age at diagnosis [6] show better prognosis. In the present case, although the patient was 90 years old, a safety margin of about 10 mm was set because of the exophilic nature of the tumor, resulting in histopathologically complete resection. In conclusion, we report a rare case of osteosarcoma arising in the mandible of a 90-year-old man. This case showed that it was not necessary to exclude surgical treatment merely because the patient was aged 90 years. Indications for surgery should be determined individually to improve the patient's quality of life.

## Data Availability

The datasets used during the current study are available from the corresponding author on reasonable request.
